# Weekly Folic Acid Is a Convenient and Well-Tolerated Alternative to Daily Dosing in Pediatric Patients with Inflammatory Bowel Disease on Methotrexate

**DOI:** 10.3390/nu15071586

**Published:** 2023-03-24

**Authors:** Tsega Adera Temtem, Maggie Vickers, John Whitworth

**Affiliations:** 1 Department of Pediatrics, Division of Pediatric Gastroenterology, Le Bonheur Children’s Hospital, University of Tennessee Health Science Center, 50 North Dunlap, Memphis, TN 38103, USA; 2 Department of Pediatrics, Division of Pediatric Gastroenterology, University of Louisville, Louisville, KY 40202, USA

**Keywords:** inflammatory bowel disease, Crohn’s disease, ulcerative colitis, methotrexate, pediatrics, folic acid, folate

## Abstract

Background: Inflammatory bowel disease (IBD) is a chronic autoimmune disorder that affects the gastrointestinal tract. Methotrexate is a folate analog immunosuppressant used in the management of pediatric IBD. Daily folic acid supplementation is currently recommended to prevent folate deficiency and reduce the side effects of methotrexate such as nausea, stomatitis, and hepatotoxicity. The aim of this study was to evaluate the safety and adequacy of once-weekly folic acid supplementation in pediatric inflammatory bowel disease patients taking methotrexate. Methods: In this single-arm observational study, we included subjects aged 2–21 years old with inflammatory bowel disease who were receiving a standard oral methotrexate dose of 10–15 mg/m^2^ weekly and 800 mcg of folic acid daily. Baseline folate level, blood counts and chemistries, and a symptom questionnaire were completed. Subjects were switched to weekly 800 mcg of folic acid to be taken in conjunction with methotrexate. Monthly phone calls with a standardized questionnaire were used to assess compliance and any change in symptoms. Follow-up blood tests were obtained 6 months after enrollment. Normal folate level was defined as >5.38 ng/mL. Results: Thirty-one subjects were enrolled. Five subjects were withdrawn due to poor compliance or transition to adult gastroenterology. Twenty-one (81%) subjects had Crohn’s disease (17 with ileal involvement) and five (19%) had ulcerative colitis. Twelve (39%) subjects were on methotrexate as a combination therapy with a biologic agent. At the 6-month follow-up visit, all subjects had stable folic acid levels (>5.38 μg/L) without macrocytic anemia. Monthly questionnaires found no increased symptoms, and there were no adverse events. Conclusions: Once weekly folic acid supplementation at a dose commonly found in a multivitamin may be sufficient to maintain normal folate levels without the development of adverse symptoms in pediatric patients with inflammatory bowel disease on methotrexate therapy.

## 1. Introduction

Inflammatory bowel disease (IBD) is a chronic, autoimmune disorder that affects the gastrointestinal tract in genetically predisposed children and adults. Inflammation from IBD, typically identified as Crohn’s disease or ulcerative colitis, can manifest with symptoms of abdominal pain, diarrhea, hematochezia, weight loss, poor growth, and/or malnutrition depending on disease location. There are several medication options for pediatric patients diagnosed with inflammatory bowel disease to induce and maintain remission, including corticosteroids, 5-aminosalicylic acid, immunomodulators, and biologic agents. Immunomodulators include azathioprine, 6-mercaptopurine, and methotrexate. Methotrexate is a folate analog used to target actively proliferating tissues in inflammatory bowel disease by decreasing pro-inflammatory cytokines and inducing lymphocyte apoptosis [1]. It can be used as a single-therapy immunomodulator in IBD or as adjunctive therapy for patients on biologic therapy to prevent antibody formation. Methotrexate is often used in patients with mild to moderate Crohn’s disease (mainly male patients due to its teratogenic effect) or in patients with thiopurine ineffectiveness or intolerance. A review on the effectiveness and therapeutic role of methotrexate in pediatric patients with IBD showed that methotrexate was effective in the maintenance of remission for 1 year in 25–69% of thiopurine-resistant and thiopurine-intolerant patients [2]. 

Patients taking methotrexate are at risk of folate deficiency due to the reversible competitive inhibition of dihydrofolate reductase (DHFR) [1]. As such, the recommendation is to provide daily folic acid supplementation to pediatric patients diagnosed with IBD who are taking methotrexate [3]. The naturally occurring form of folic acid has a reduced form, known as folate [4]. Folate is a water-soluble B vitamin that is essential for normal cell growth and replication, and its main role is to prevent DNA damage and cell injury [5]. The human body is unable to produce folate, so it must be supplemented in the synthetic form of folic acid either through a traditional or fortified diet or by means of oral supplementation [4]. Folate is primarily absorbed in the duodenum and proximal jejunum [4]. Folic acid supplementation not only helps to prevent folate deficiency, but also to reduce the adverse side effects of methotrexate, such as nausea, stomatitis, and hepatotoxicity [5]. While the optimal dose of folic acid has not been established, the recommended frequency is daily supplementation of folic acid [3]. Some researchers have suggested weekly folic acid dosing in adults with rheumatoid arthritis who are taking methotrexate, but the effectiveness of vitamin supplementation at this frequency was not assessed [6,7,8]. Establishing the safety and adequacy of weekly dosing may provide benefits, as surveys of adolescents with inflammatory bowel disease report difficulty with adherence to daily multi-drug regimens [9,10]. Thus, providing simultaneous administration of medications, such as a single weekly dose of folic acid for patients taking once weekly methotrexate, may help improve compliance and health-related quality of life. 

The aim of this study was to evaluate the safety and adequacy of once-weekly folic acid supplementation in pediatric inflammatory bowel disease patients taking methotrexate. We aimed to assess this by using subjective reports of patient adherence and symptoms in combination with objective findings of laboratory changes.

## 2. Materials and Methods

### 2.1. Study Design

This prospective single-arm observational study was conducted from September 2018 to June 2019 at a tertiary care pediatric hospital. Inclusion criteria required that all subjects have an underlying diagnosis of either Crohn’s disease or ulcerative colitis. Additionally, patients were required to be on standard oral methotrexate dosing of 10–15 mg/m^2^ weekly and daily folic acid supplementation. Exclusion criteria included age less than 2 years old or greater than 21 years old or an underlying diagnosis of inflammatory bowel disease undetermined (IBD-U). Patients were also excluded if they had an abnormal baseline folate level or evidence of macrocytosis on complete blood count panel. Lastly, subjects with a history of prior bowel resection were excluded as this is an independent risk factor for folate deficiency, and the recommended timelines for disease surveillance do not apply to patients with extensive bowel resection [11]. Furthermore, subjects taking any folate antagonists other than methotrexate were not eligible for this study. 

### 2.2. Outcome Measures: Demographics

Electronic medical records were used to screen for subject eligibility based on the above criteria. Demographic and clinical information were obtained from the medical record including sex, age, race, underlying diagnosis (Crohn’s disease or ulcerative colitis), disease duration, and current medications. Of the 250 pediatric inflammatory bowel disease patients identified, 40 patients met all criteria for inclusion. The minimum duration of enrollment for each subject was 6 months. Subjects could be withdrawn from the study due to the development of abnormal folate levels, poor medication compliance, new or unexplained symptoms, or discontinuation of methotrexate.

### 2.3. Outcome Measures: Laboratory Markers 

The serum folate level was obtained at the onset of the study, in addition to a complete blood count panel, transaminases, and inflammatory markers. Prior to transitioning to weekly dosing of folic acid, patients had to have a normal folate level (>5.38 ng/mL) and normocytic mean corpuscular volume (80–100 fL). If patients had an abnormal baseline folate level or MCV, they were subsequently excluded from further participation in this study. Laboratory testing was repeated 6 months after enrollment. Serum folate testing was included in our routine laboratory analysis. Additional testing with red blood cell (RBC) folate concentration and vitamin B12 was reserved for subjects who developed macrocytosis (>100 fL) or symptoms despite normal folate levels at any point during the study [12].

### 2.4. Outcome Measures: Symptom Reporting

Subjects completed a questionnaire at enrollment to assess baseline symptoms, including the presence of oral ulcers/stomatitis, nausea, abdominal pain, diarrhea, fatigue, headaches, and difficulty with concentration. They received monthly phone calls to identify any unexplained or new symptoms, evaluate for any change in baseline symptoms, and assess medication adherence. Medication adherence was assessed through discussion with the patient or caregiver and with further questioning to confirm medication dose and frequency. Monthly phone calls were standardized and included the same questions asked in the baseline questionnaire to evaluate for any new, or changes to, frequency of mouth ulcers, abdominal pain, nausea, diarrhea, headache, irritability, or difficulty concentrating. Two investigators alternated these phone calls to limit bias. Furthermore, the questionnaire was in a “yes/no” format to minimize bias and subjectivity. The questionnaire was considered positive if the patient answered yes to having developed any new symptoms (mouth ulcers, abdominal pain, nausea, diarrhea, headache, irritability, difficulty concentrating) that were increased from the original baseline questionnaire. Phone call questionnaires were completed by the patient or caregiver.

### 2.5. Outcome Measures: Supplementation 

Once enrolled, subjects were switched to once weekly dosing of 800 mcg of folic acid to be taken on the same day as their methotrexate. Folic acid was taken either as an individual supplement or within a multivitamin. Folic acid supplements are available in doses of 400 mcg, 800 mcg, and 1 mg. We selected 800 mcg as our standard dose as this is a commonly accessible amount in a multivitamin, and several of our patients preferred to continue some form of multivitamin supplementation during the study. If a particular subject preferred to take a multivitamin rather than a separate folic acid supplementation, the specific amount of folic acid within the multivitamin was confirmed to be 800 mcg, or they were switched to a different multivitamin with this exact amount of folic acid. Therefore, regardless of whether a patient elected to take a multivitamin with folic acid or regular folic acid supplementation, the amount of folic acid (800 mcg) was still the same among all participants. 

### 2.6. Safety Measures

As a safety measure, there was a plan in place for any subjects reporting new or increased symptoms on the monthly questionnaire to return earlier than the scheduled appointment for clinical and laboratory evaluation. Since none of the participants had reports of an increase in symptoms from baseline, earlier clinic visits or laboratory evaluations were not implemented.

### 2.7. Clinical Outcomes

We measured the clinical outcome as the maintenance of folate levels at or above baseline levels for each patient after switching from daily to weekly folic acid supplementation. The secondary clinical outcome measure was the development of adverse symptoms after switching to weekly dosing of folic acid supplementation.

### 2.8. Statistical Analysis

Statistical analysis was performed by the University of Tennessee Health Science Center Biostatistics, Epidemiology, and Research Design (BERD) Center. Frequency tables were used to assess study variables of interest, including sex, race, disease involvement, and biologic therapy. Pre- and post-study labs were compared based on diagnosis by calculated mean and standard deviation. *t*-tests with box plots were used to compare pre- and post-study lab results, including folate, hemoglobin, hematocrit, MCV, liver transaminases, and inflammatory markers. Linear regression models were used to assess the effects of biologic therapy, disease location, duration of disease, race, and age on the change in folate level from the beginning to the end of the study. 

### 2.9. Ethical Statement

The study was approved by the University of Tennessee Institutional Review Board which provided ethical approval on 16 April 2018 for this study, IRB number 18-05808-FB. All procedures performed in studies involving human participants were in accordance with the ethical standards of the institutional research committee and with the 1964 Helsinki declaration and its later amendments or comparable ethical standards. 

## 3. Results

### 3.1. Study Population

**Figure 1:**
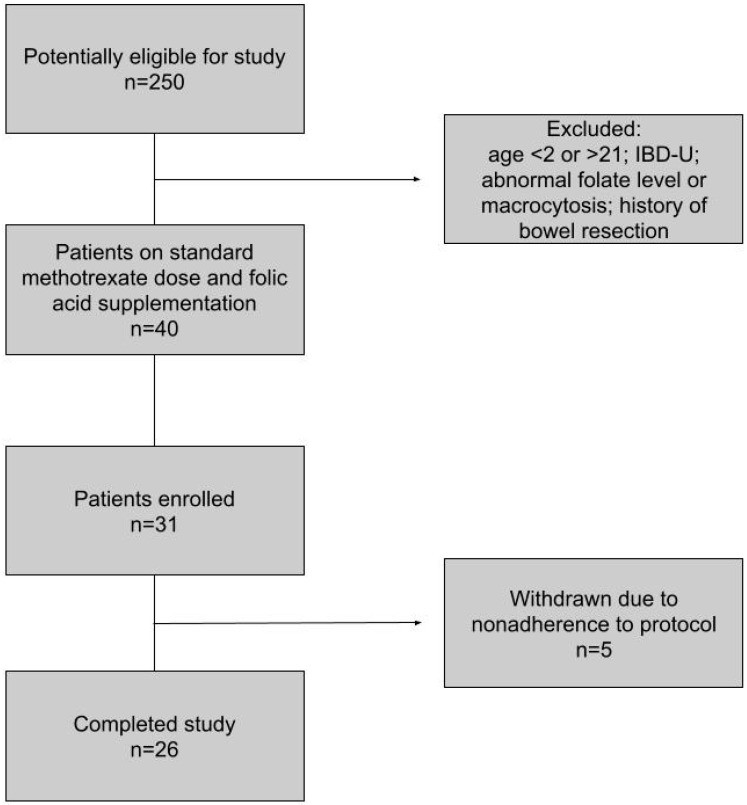
Flowchart of the study.

Of the 40 patients meeting the inclusion criteria, 31 patients consented and were enrolled, with ages ranging between 7 and 21 years old (Figure 1). Of the 31 subjects enrolled, 26 completed the study with 100% adherence. Five patients were withdrawn for reasons including poor medication adherence on monthly questionnaires, missing clinic appointments with resultant protocol deviation, or transition to adult gastroenterology during the study. None of the subjects were withdrawn due to symptoms or abnormal folate levels. Of those who completed the study, 21 (81%) had Crohn’s disease (17 with ileal involvement) and five (19%) had ulcerative colitis; 25 (96%) were male (due to the general avoidance of methotrexate in females given teratogenicity). The mean age of patients enrolled was 15.2 years old (standard deviation (SD) = 3.1 years). Of those patients with Crohn’s disease, the average duration of disease since diagnosis was 3.1 years (SD = 2.6 years). Patients diagnosed with ulcerative colitis had a slightly longer average disease duration of 4.2 years (SD = 4.9 years). There were 12 (46.2%) subjects who were on methotrexate as a combination therapy with a biologic (adalimumab or infliximab). Demographic and selected clinical features of the study population were assessed (Table 1). 

### 3.2. Folate Level and Associations

**Figure 2:**
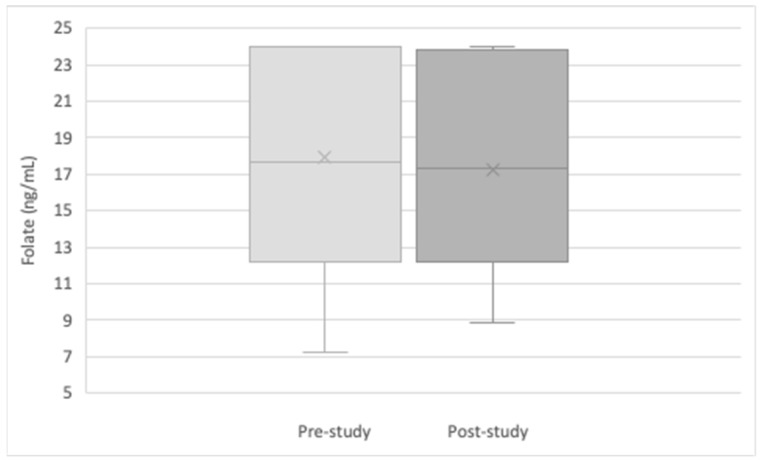
Pre- to post-study difference in folate (ng/mL).

**Figure 3:**
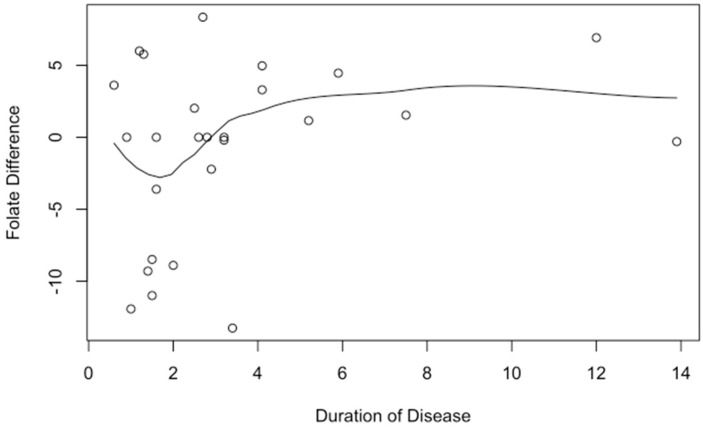
Folate difference pre- to post-study by Duration of Disease.

**Figure 4:**
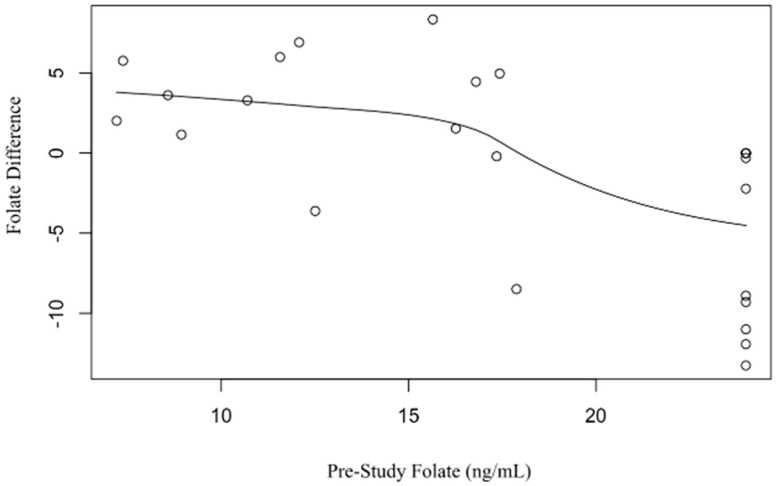
Baseline folate level (ng/mL) predicting pre- to post-study folate difference.

All patients had a normal folate level (>5.38 ng/mL) and normocytic mean corpuscular volume (MCV) (80–100 fL) at the beginning of the study before transitioning to weekly dosing of folic acid. There was no difference in folate levels of patients taking daily versus weekly doses (Mean Difference (MD) 0.81 ng/mL, *p* = 0.51), which confirmed the study hypothesis (Figure 2). We compared folate differences from pre- to post-study with several demographic and disease characteristics including the following: disease location (small bowel versus large bowel versus both), disease duration from time of initial diagnosis, age, race, and use of biologics. None of these met statistical significance. Although not statistically significant, there were several trends toward a greater difference in folate level from pre- to post-study: biologic therapy, small bowel disease involvement, increased duration of disease, and younger age. Each year of prior disease duration of inflammatory bowel disease was associated with a greater difference in folate level from pre- to post-study (*p* = 0.019) (Figure 3). Each year of age was associated with a lower folate difference between pre- and post-study folate levels (*p* = 0.018). The effect of pre-study folate levels in comparison to post-study folate levels was significant (*p* = 0.001). Patients with a higher baseline folate level were found to have less of a difference in folate level from pre- to post-study. Furthermore, each one-point increase in pre-study folate levels corresponded with a lower folate difference by −0.58 (Figure 4), indicating that there was less of a change between pre- and post-study folate in those patients with a higher initial baseline folate level.

### 3.3. Laboratory Test Results

Additional laboratory values from pre- to post-study were compared including hematocrit, mean corpuscular volume (MCV), erythrocyte sedimentation rate (ESR), and alanine transaminase (ALT). Specific graphs and *p* values for each comparison were analyzed (Supplementary Materials, Figure S1a–d). Pre- to post-ALT was the only laboratory value that showed significance (MD = 11.6 IU/L, *p* = 0.002); however, this was a negative correlation and did not appear to bear any clinical significance. There was no difference from pre- to post-study in hematocrit (MD = −0.66%volume, *p* value = 0.24), MCV (MD = 0.35 fL/cell, *p* value = 0.58), and ESR (MD = 2.6 mm/h, *p* value = 0.2).

### 3.4. Symptom Questionnaires

Monthly phone call questionnaires of the 26 subjects showed no new or unexplained symptoms from baseline during the 6-month time period. The most common symptoms reported on the baseline questionnaire were neurologic symptoms, including headache, irritability, fatigue, or difficulty concentrating. Patients diagnosed with Crohn’s disease had more reported symptoms at baseline compared to those with ulcerative colitis.

## 4. Discussion

Overall, this study showed that in a small pediatric population with inflammatory bowel disease taking methotrexate, there was no difference in folate levels in patients taking daily versus weekly doses (Mean Difference (MD) 0.81 ng/mL, *p* = 0.51) over a 6 month time interval. Furthermore, weekly folic acid supplementation seems to be well tolerated, as patients did not report any increase in symptoms on the monthly questionnaire compared to the baseline survey. Evaluation of weekly folic acid supplementation has not been previously studied in pediatric patients with inflammatory bowel disease on methotrexate, and the current practice in pediatrics remains daily folic acid supplementation in patients with inflammatory bowel disease on methotrexate [3]. However, several studies have evaluated different dosages and frequencies of folic acid in adult patients with rheumatoid arthritis. One study found no benefit in symptom reduction or laboratory abnormalities (transaminitis or cytopenias) when comparing a higher dose of folic acid (30 mg per week) to lower folic acid dosing (10 mg per week) in patients with rheumatoid arthritis with an average of 21–22 mg/week of methotrexate; however, change in folate level was not assessed [6]. Similarly, another study showed no significant difference in the efficacy of methotrexate when comparing 5 mg weekly to 27.5 mg weekly of folic acid supplementation [7]. A more recent study in rheumatoid arthritis patients taking 20 mg of methotrexate weekly showed no significant difference in methotrexate toxicity or disease activity between patients taking 5 mg/week and those taking 0.8 mg/week of folic acid [8]. The primary aim of our study was to compare the frequency of folic acid administration; therefore, we maintained all patients on a standard dose of 800 mcg of folic acid, as we felt that variations in dosing would confound the results. Similar to the adult studies in patients with rheumatoid arthritis taking methotrexate, there were no adverse symptoms from weekly supplementation of folic acid. In contrast to these studies, our study provides additional supporting evidence with stable folate levels. 

Adherence to a multi-drug regimen is difficult in patients with chronic diseases, with studies showing non-adherence rates up to 93% in adolescents with inflammatory bowel disease [10]. In a study by Speckhorst et al., adolescents with chronic diseases reported more difficulty with medication adherence than those with monotherapy, with an associated decrease in quality of life, difficulty with coping strategies, and anxiety [10]. Patients with inflammatory bowel disease are often taking several medications, some of which are added to reduce the side effects of primary therapy, such as folic acid supplementation while taking methotrexate or acid reducers for those on steroids. Folic acid supplementation is recommended to reduce the toxic effects of methotrexate, although the optimal dose has not been well established and remains controversial.

Guidelines recommend measuring serum folate levels at least annually, or if macrocytosis is present [13]. Patients at higher risk of folate deficiency were excluded from our study, specifically those with a history of small bowel resection, as recommended timelines do not apply to these patients and they often need closer and more frequent monitoring [11]. In our study, hematocrit and mean corpuscular volume (MCV) were obtained at enrollment and again at 6 months to screen for macrocytosis (MCV > 100), which would suggest possible folate or vitamin B12 deficiency. None of our patients had macrocytosis at baseline or on repeat laboratory evaluation. Additionally, pre- to post-study MCV was stable (MD = 0.35 fL/cell, *p* = 0.58).

Red blood cell (RBC) folate concentration was not used routinely in our study as it would not accurately reflect recent folate changes in symptomatic patients. Serum folate was utilized in this study as it is more cost-effective, correlates better with homocysteine levels, and more accurately reflects recent changes in folate status [14,15,16]. RBC folate responds more slowly to changes in intake [17]. While it can provide an index of long-term folate status, it can be falsely low in vitamin B12 deficiency, as the latter is required for the uptake of 5-methylTHF into red blood cells [15]. This is an especially important consideration in our study as patients with Crohn’s disease and ileal involvement are at increased risk of vitamin B12 deficiency. Thus, we utilized serum folate levels in accordance with ECCO (European Crohn’s and Colitis Organization) guidelines [13]. However, in patients with normal serum folate values but who had a high clinical suspicion for folate deficiency based on symptoms or macrocytosis, our protocol was to also measure red blood cell folate and vitamin B12 levels [12]. We did not have to implement this protocol in our study as none of the patients developed an abnormal folate level, macrocytosis, or clinical symptoms concerning for folate or B12 deficiency.

The etiology of folate deficiency in pediatric patients with IBD may be multifactorial, including low dietary intake, malabsorption, active inflammatory state, and medications [18]. The amount of dietary folate intake was not specifically monitored in this study. Studies in methotrexate-treated patients with rheumatoid arthritis show that supplementation with folic acid reduces the incidence of adverse gastrointestinal effects and hepatic dysfunction, as measured by elevation in transaminases [19]. We used an increase in symptoms from baseline or unexplained new symptoms as an indirect measure of inadequate folic acid supplementation. Due to limited total body stores of folate, dietary restriction can result in a deficiency state within two months [14], and thus the standardized telephone questionnaire was performed monthly as a safety measure. Evaluation of serum folate reflects more recent changes in intake [14,15], so this level was obtained at enrollment to provide a baseline comparison in the event of patients developing symptoms during the study. The protocol for patients with new or increased symptoms on the questionnaire was for them to return for an earlier clinic visit including repeat laboratory assessment due to concern for possible side effects from methotrexate due to inadequate folic acid supplementation. None of our patients developed increased symptoms during the study or had to obtain bloodwork sooner than the 6-month scheduled follow-up.

Cobalamin (vitamin B12) and folate deficiency can occur in patients with IBD independent of drug-induced deficiencies. This can occur particularly after ileal resection (>20 cm terminal ileal resection) or in those with extensive ileal inflammation. These vitamin deficiencies are rare in pediatric patients diagnosed with inflammatory bowel disease. Folate deficiency is more common in pediatric patients with Crohn’s disease compared to ulcerative colitis, with a reported frequency of 10–13% in Crohn’s disease compared to 3.8–9.7% in ulcerative colitis [18,20]. Of the 21 patients with Crohn’s disease who completed our study, 17 patients had small bowel involvement at diagnosis. None of these patients developed clinical or laboratory evidence of folate deficiency; however, all of our patients who participated were noted to be in clinical remission during the study. Although not significant, it is interesting to note that patients with small bowel disease involvement did have a larger difference in folate level between pre- and post-study (*p* = 0.92), which could play a role in patients who are expected to be on methotrexate longer than 6 months.

### 4.1. Limitations

Some limitations include the single-arm study design that lacks a concurrent comparison group and the observational nature that limits our ability to deduce efficacy. Furthermore, our population focused on a specific subset of pediatric patients which subsequently limited the sample size, and thus was not optimal for randomization to a control arm.

A further limitation was the study duration. Methotrexate can be used as the primary maintenance therapy in IBD or as an adjuvant therapy with a biologic to prevent antibody formation. When used in combination with a biologic, recent studies suggest that six months is the optimal time to achieve a benefit, after which the immunomodulator can be discontinued [21]. Subsequently, subjects in our study were enrolled for a minimum of 6 months, after which almost half of them no longer required methotrexate or folic acid supplementation. Future studies are needed to ensure that an adequate folate level is maintained for IBD patients who require methotrexate for longer than 6 months.

At our institution, methotrexate is seldom used in females of childbearing potential due to teratogenicity. As a result, the vast majority of patients enrolled in the study were male, which limits the generalizability of the results.

While we standardized the dosing of methotrexate and the folic acid supplementation in our study, we did not account for variations in dietary sources of folate. The impact of this is likely very small, and future studies may consider incorporating dietary records, such as a daily food diary.

A further limitation of the study is that the questionnaires were administered by study personnel, which could introduce investigator bias. This risk was decreased by alternating investigators each month. Future studies could utilize online questionnaires or surveys for patients to complete to further limit subjectivity or investigator bias.

### 4.2. Further Research

While the focus of our study was to evaluate the safety of weekly folic acid administration, further studies on dose selection in the presence of active small bowel disease may be beneficial. We used 800 mcg in this prospective study for ease of dosing, but higher weekly doses with more frequent monitoring may be considered in clinical practice depending on disease involvement and provider or patient preference. Additionally, large multicenter studies may be beneficial, as a larger sample size may allow for randomization to a control arm and further strengthen these results, as it would be possible to compare results from a control group taking daily folic acid supplementation to another group taking weekly folic acid supplementation.

## 5. Conclusions

In conclusion, once-weekly folic acid supplementation at a dose available in a multivitamin may be a safe and well-tolerated alternative to daily dosing for children with inflammatory bowel disease who are taking methotrexate. This study demonstrated maintenance of normal folate levels without the development of adverse side effects for at least 6 months in a subset population of pediatric patients with inflammatory bowel disease on maintenance therapy with methotrexate. This may provide a benefit and improvement in medication adherence in patients on multiple medications for the management of inflammatory bowel disease.

## Figures and Tables

**Table 1:** Demographics and selected clinical features of the study population.

Characteristic	Value (N = 26), n (%)
**Sex**	
Male	25 (96%)
Female	1 (4%)
**Race**	
White	19 (73%)
African American	6 (23%)
Hispanic	1 (44%)
**IBD type**	
Crohn’s disease	21 (81%)
Ulcerative colitis	5 (19%)
**Disease involvement**	
Large bowel	8 (31%)
Small bowel	6 (23%)
Small and large bowel	11 (42%)
**Biologic therapy**	12 (46%)

## Data Availability

The data presented in this study are available upon request from the corresponding author.

## Supplementary Materials

The following supporting information can be downloaded at: https://www.mdpi.com/article/10.3390/nu15071586/s1, Figure S1: Pre- and post- study levels of (a) hematocrit, (b) mean corpuscular volume, (c) erythrocyte sedimentation rate, (d) alanine aminotransferase.
