# Supplementary material for: Weekly Folic Acid Is a Convenient and Well-Tolerated Alternative to Daily Dosing in Pediatric Patients with Inflammatory Bowel Disease on Methotrexate
Source: Nutrients. 2023 Mar 24;15(7):1586. doi: 10.3390/nu15071586 (PMC10096625; doi:10.3390/nu15071586)
Supplement: Supplementary file 1 [file nutrients-15-01586-s001.zip › nutrients-2269952-supplementary.pdf]

Supplementary Materials:

Figure S1: Pre- and post- study levels of (**a**) hematocrit, (**b**) mean corpuscular volume, (**c**) erythrocyte sedimentation rate, (**d**) alanine aminotransferase.

(**a**)

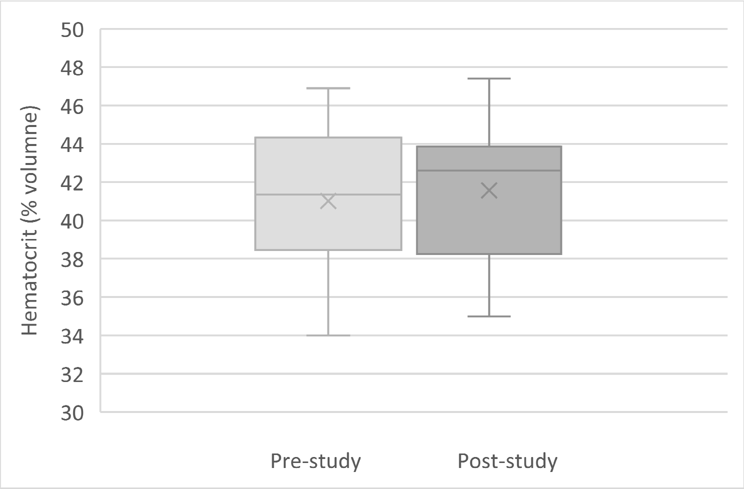

(**b**)

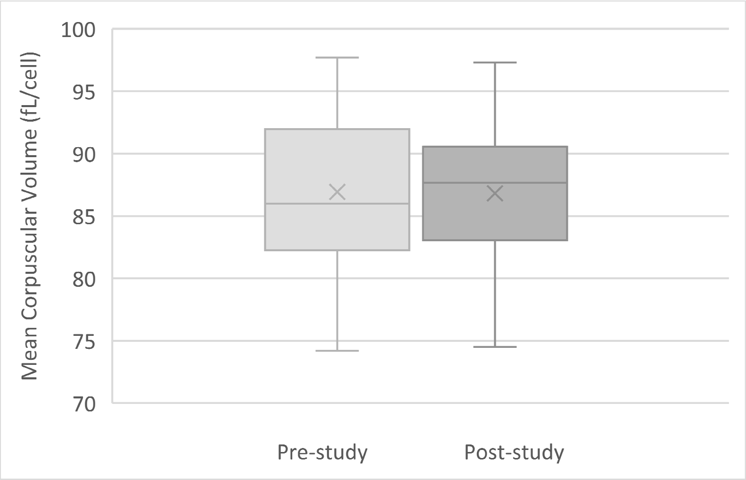

(**c**)

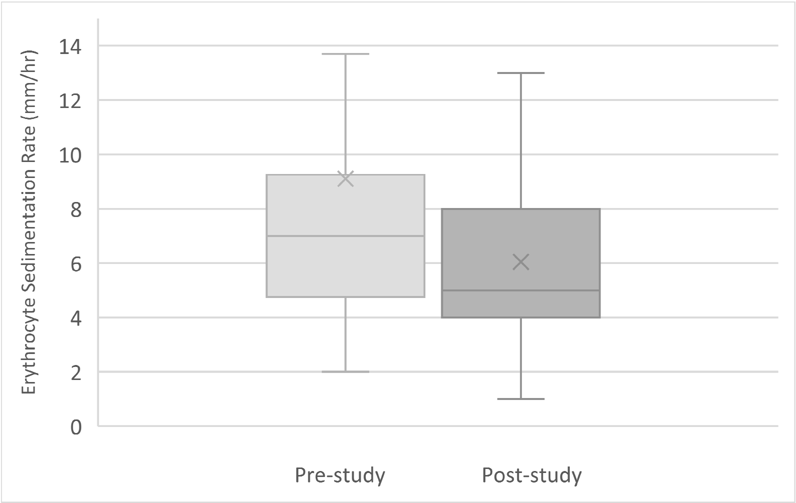
Erythrocyte Sedimentation Rate (mm/hr)
14
12
10
8
6
4
2
0
Pre-study
Post-study

(**d**)

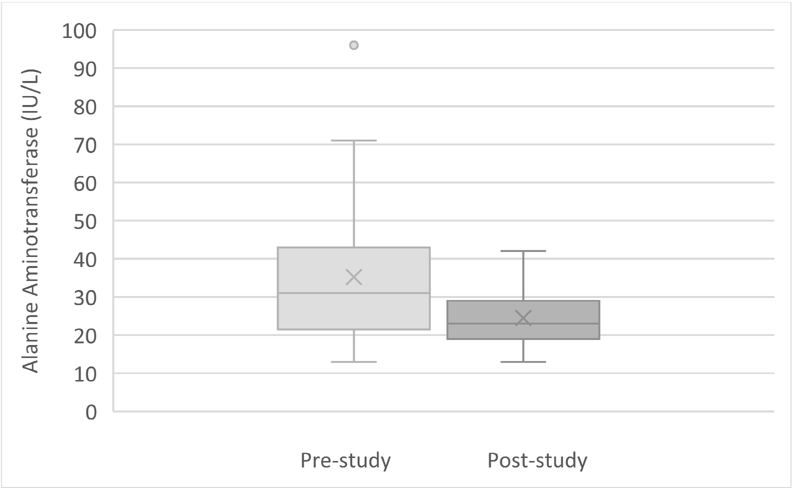
Alanine Aminotransferase (IU/L)
100
90
80
70
60
50
40
30
20
10
0
Pre-study
Post-study
